# Nosocomial Transmission of *C. difficile* in English Hospitals from Patients with Symptomatic Infection

**DOI:** 10.1371/journal.pone.0099860

**Published:** 2014-06-16

**Authors:** Esther van Kleef, Antonio Gasparrini, Rebecca Guy, Barry Cookson, Russell Hope, Mark Jit, Julie V. Robotham, Sarah R. Deeny, W. John Edmunds

**Affiliations:** 1 London School of Hygiene and Tropical Medicine, London, United Kingdom; 2 Public Health England, Colindale, London, United Kingdom; 3 University College London, London, United Kingdom; Institute Pasteur, France

## Abstract

**Background:**

Recent evidence suggests that less than one-quarter of patients with symptomatic nosocomial *Clostridium difficile* infections (CDI) are linked to other in-patients. However, this evidence was limited to one geographic area. We aimed to investigate the level of symptomatic CDI transmission in hospitals located across England from 2008 to 2012.

**Methods:**

A generalized additive mixed-effects Poisson model was fitted to English hospital-surveillance data. After adjusting for seasonal fluctuations and between-hospital variation in reported CDI over time, possible clustering (transmission between symptomatic in-patients) of CDI cases was identified. We hypothesised that a temporal proximity would be reflected in the degree of correlation between in-hospital CDI cases per week. This correlation was modelled through a latent autoregressive structure of order 1 (AR(1)).

**Findings:**

Forty-six hospitals (33 general, seven specialist, and six teaching hospitals) located in all English regions met our criteria. In total, 12,717 CDI cases were identified; seventy-five per cent of these occurred >48 hours after admission. There were slight increases in reports during winter months. We found a low, but statistically significant, correlation between successive weekly CDI case incidences (phi = 0.029, 95%CI: 0.009–0.049). This correlation was five times stronger in a subgroup analysis restricted to teaching hospitals (phi = 0.104, 95%CI: 0.048–0.159).

**Conclusions:**

The results suggest that symptomatic patient-to-patient transmission has been a source of CDI-acquisition in English hospitals in recent years, and that this might be a more important transmission route in teaching hospitals. Nonetheless, the weak correlation indicates that, in line with recent evidence, symptomatic cases might not be the primary source of nosocomial CDI in England.

## Introduction

*Clostridium difficile* infection (CDI) is a source of considerable morbidity and mortality for hospitalised patients, and its prevention, control and treatment place a substantial burden on healthcare systems[1], [2]. Since 2007, in addition to improved antimicrobial stewardship and mandatory surveillance, enhanced infection control measures to prevent *C. difficile* transmission have been implemented in England. These measures have focused on isolating symptomatic patients and improving hospital-cleaning regimens, with the goal of meeting government-led CDI reduction targets. Reported cases of CDI have dropped from 55,498 in 2007/08 to 18,005 in 2011/12[3], at a time when the prevalence of the hyper-virulent *C. difficile* BI/NAP1/027 also decreased[4]. Apart from improved antimicrobial stewardship, guidelines for CDI prevention and control assume that symptomatic patients in hospitals account for most *C. difficile* transmission and consequent infection (CDI). However, in 2012 and 2013, research using whole genome sequencing of hospital and community isolates from Oxfordshire, United Kingdom, has challenged this assumption. Eyre *et al* found a high level of genomic diversity in samples from symptomatic CDI patients. Moreover, only a minority of hospital-onset cases of CDI were found to share an epidemiological link as well as genomic link with a symptomatic CDI case[5]–[7].

This recent evidence was limited to a small sample of hospitals that were all located in one English county. To explore whether these new developments in our understanding of the epidemiology of CDI are more generally applicable, we investigated the presence of clustering in symptomatic CDI patients, indicative of patient-to-patient *C. difficile* transmission, in a wide range of hospitals in England between 2008 and 2012.

## Methods

### Data

The dataset consisted of mandatory reported details of each identified CDI case >2 years of age collected from all 167 National Health Service (NHS) Trusts via a web-enabled surveillance system, held by Public Health England (PHE)[8]. Details included the dates of admission and faecal sampling, patient category (e.g. inpatient, outpatient etc.) and age. Data covering the period between April 2008 and March 2012 were extracted from this surveillance scheme. To ensure consistency in the reported observations, we restricted our analyses to NHS acute trusts that followed the Department of Health's CDI testing guidance according to a survey held in 2010[9]. In England, a two test screening algorithm has been advocated and hospital trusts are recommended to test patients with diarrhoea (Bristol Stool Chart types 5–7)[10] using either a GDH Enzyme Immunoassay (GDH EIA), a nucleic acid amplification test (NAAT) or the Polymerase Chain Reaction (PCR), followed by a toxin sensitive EIA (or a cell cytotoxin neutralisation assay). If both the first test and the second test are positive, the case is eligible for reporting to PHE[11]. This resulted in the selection of data from 46 hospitals, belonging to 28 individual NHS acute Trusts, and excluded any of the Oxfordshire hospitals (see *table 1*). Only CDI positive in-patients were included for analysis (i.e. excluding regular attendees, outpatients and patients having visited only accident and emergency departments). In order to evaluate healthcare facility associated infections, patients with onset of symptoms <48 hours after admission were excluded[12]. We aggregated the reported data per hospital by week, using the date of faecal sampling as the time of onset of CDI related symptoms.

**Table 1:** Description of CDI data from 46 selected hospitals.

	N	Median/Mean for 4 year period	IQR (Q_1_-Q_3_)	Median/Mean p.w.	IQR (Q_1_-Q_3_)	Cases per 10,000 bed-days available (mean/median)
**Number of weeks**	**209**	**-**	**-**	**-**	**-**	**-**
**Beds available per hospital**	**-**	**422/423**	**243–515**	**-**	**-**	**-**
* General (n = 33)*	-	444/416	346–500	-	-	-
* Teaching (n = 6)*	-	837/754	799–941	-	-	-
* Specialist (n = 7)*	-	134/169	95–243	-	-	-
**CDI cases reported**	**12,717**	**244/276**	**138–377**	**1/1.3**	**0-2**	**4.1/4.6**
* General*	8,974 (70.6%)	253/272	194–352	1/1.3	0-2	4.1/4.5
* Teaching*	3,348 (26.3%)	551/558	331–690	2/2.7	1-4	5.6/6.4
* Specialist*	395 (3.1%)	37/56	30–77	0/0.27	0-0	1.8/3.1
**CDI cases reported with onset >48 h**	**9,574**	**184/208**	**104–270**	**1/1.0**	**0-1**	**3.1/3.5**
* General*	6,779 (70.8%)	200/205	140–247	1/1.0	0-2	3.2/3.5
* Teaching*	2,504 (26.2%)	370/417	252–534	1/2.0	0-3	4.1/5.3
* Specialist*	291 (3.0%)	31/42	28–53	0/0.2	0-0	1.2/2.2

Summary statistics of CDI cases reported to the English mandatory surveillance system by a selection of 46 hospitals from the period of April 2008 to March 2012. IQR = Interquartile range; p.w. = per week.

### Statistical methods

**Figure 1:**
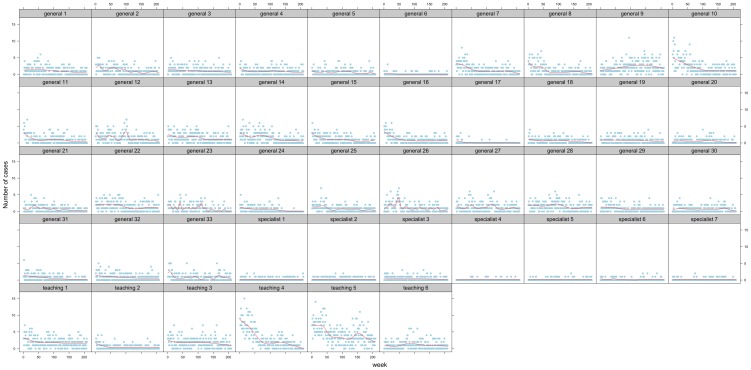
Observed weekly number of CDI per hospital over the four-year study period. Grey dots represent the weekly-observed CDI cases within all hospitals from April 2008 to March 2012. X-axis: Week 0 corresponds to the first week of April 2008 and week 209 to the last week of March 2012. Red line: the incidence trend over time illustrated by cubic smoothing spline fit (for illustration).

A generalized additive mixed-effects Poisson model, allowing for overdispersion, with a log link[13], [14], was used for the weekly observations of CDI counts. Three effects were identified that required inclusion in the linear predictor of this model. Firstly, hospital was introduced as a categorical variable to allow for potentially strong clustering due to differences in size, case-mix, and region (*see* *table 1*). Secondly, a fixed polynomial-by-hospital interaction term was included to accommodate varying rates of change (primarily decline) over the four-year period in observed CDI per hospital; *Figure 1* shows the time series of symptomatic CDI per hospital. Thirdly, a cyclic effect was included using a periodic penalised cubic regression spline to accommodate seasonal patterns of CDI, as have been observed previously in settings outside England, and which have been attributed to increased levels of “at risk" antibiotic use (e.g. ciprofloxacin) during the winter months (January to March), and influenza (which can lead to secondary bacterial infections requiring antibiotic treatment)[15]–[17]. The intention was that these three terms would account for the longitudinal behaviour of weekly CDI counts. Finally, a random error term was added to the linear predictor with an autoregressive correlation structure of order 1 (AR(1)) that would accommodate local (in time) departures from this base model. The autoregressive component of this error would be an indicator of local statistical dependence, and its presence would serve as a proposed marker for transmission between symptomatic cases (either directly, or indirectly via the hands of healthcare works or hospital surfaces contaminated by symptomatic cases). Full details of the model are provided in the material S1.

All analyses were performed with R 3.0.1 (Team R Development Core, website: http://cran.r-project.org/) using the R package *mgcv* [18] and *splines*. To account more accurately for the decline in observed CDI since 2007, the comparative fit of three polynomials, linear, quadratic and cubic was assessed using Akaike Information Criterion (AIC). We added a cyclic (periodic) penalised cubic regression spline over the variable week of the year and compared model fit with and without this smoothing term representing seasonal variations, again based on AIC (see *table S1*). The standardized residuals were examined for significant departures from normality[19]. In addition, the Box-Pierce portmanteau statistic was used to indicate serial dependence.

## Results

### Descriptive statistics

The 46 hospitals reported 12,717 CDI cases in the four-year study period, of which 9,574 (75.3%) had an onset >48 hours after admission. Between 2008/09 and 2009/10 there was a 30.6% decline in CDI reported from these healthcare facilities, in comparison to 20.9% (2009/10 to 2010/11) and 15.7% (2010/11 to 2011/12) in the years thereafter. This is in line with national figures (29.1%, 18.0% and 17.1% respectively). Teaching hospitals reported the highest number of cases, which did not change once adjusted for their larger hospital size (expressed in the median number of cases per 10,000 bed-days available, where available bed-days is a crude estimate of the number of hospital beds in 2013[20] multiplied by the number of days covered by the study, *see* *table 1*).

### Base-model assuming no transmission patterns

**Figure 2:**
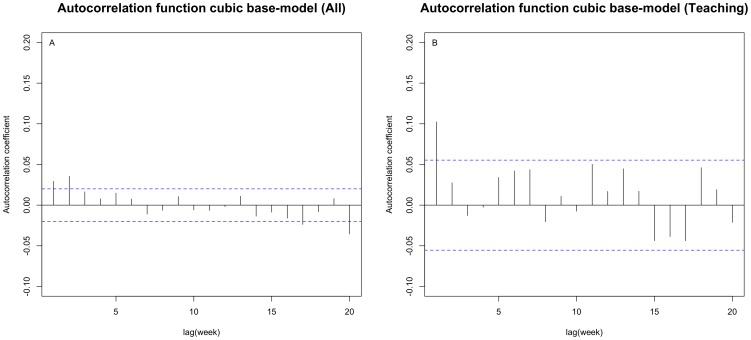
Dependence between observed weekly number of CDI – base-model. A and B: autocorrelation function (ACF) of normalized residuals of the base-model fitted to data of all hospitals (**A**) and of teaching hospitals only (**B**) including a fitted cubic representation of the CDI trend over time and seasonality. The blue lines correspond to the threshold for significance of correlation (dependence) (p<0.05) between lagged weekly observations up to week 20. E.g. crossing of this threshold by the base-model residuals at lag 1 and lag 2 for the model fitted to all hospitals suggests that a correlation exists between the observed CDI in a given week and the number of CDI cases present in the hospital one and two weeks earlier.

For all three representations of the base-model, a model including seasonal patterns provided a moderately better fit, and a combination of seasonality and a cubic time trend proved the best model fit (see table S1). By examining the correlogram of the final base-model's normalized residuals, we could identify whether there was evidence of serial dependence (*see* *figure 2A*). Such dependence could be explained by transmission between symptomatic CDI carriers. *Figure 2A* illustrates a low but significant correlation between cases in a given week and symptomatic carriers present in the hospital one and two weeks earlier (p<0.05), with a slightly stronger correlation at two weeks. Taking a total of a 20-week interval (as transmission events between hospital cases with an onset further than 20 weeks apart is assumed to be unlikely), the model revealed a highly significant Box-Pierce Q-statistic (X^2^ = 54.59, degrees of freedom (df) = 20, p = 0.00005), indicating non-independence. Therefore, the AR(1)-model was fitted, with the best fitting cubic polynomial to represent the decline in CDI over time, as well as seasonality.

### AR(1)-model assuming transmission patterns

**Figure 3:**
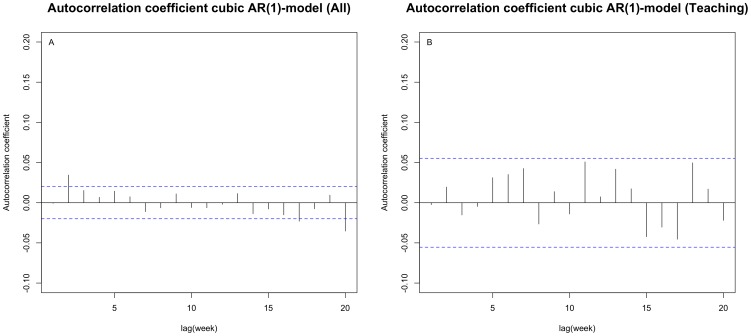
Dependence between observed weekly number of CDI – AR1-model. A and B: autocorrelation function (ACF) of normalized residuals of the AR1-model fitted to data of all hospitals (**A**) and of teaching hospitals only (**B**) including a fitted cubic representation of the CDI trend over time and seasonality. As in figure 2, the blue lines correspond to the threshold for significance of correlation (dependence) (p<0.05) between lagged weekly observations up to week 20. Crossing of this threshold by the AR1-model residuals at lag 2 suggests the AR1 structure (symptomatic cases primarily cause acquisition of *C. difficile* among patients admitted to hospital up to one week later and, to a lesser extent, to cases admitted beyond this time), does not fully explain the dependence structure between weekly observations.

*Figure S1* presents the seasonal variation of CDI in hospitals within our sample fitted by the AR(1)-model, and shows a slight increase during the months January to March. Assuming that symptomatic cases primarily caused acquisition among patients admitted to hospital up to one week later and, to a lesser extent, to cases admitted beyond this time (i.e. the AR-1 structure), the estimated magnitude of dependence was low, but statistically significant (Φ = 0.029 (95% CI = 0.009-0.049). This suggested that transmission between symptomatic CDI cases was affecting the weekly-observed CDI, but that its role in acquisition might be limited. This transmission pattern between observed weekly CDI was not fully explained by the AR(1) structure, as is indicated by the significant correlation at lag(week) 2 still being present after having fitted the AR(1) covariance structure (Box-Pierce Q-statistic (X^2^ = 44.4, df = 20, p-value = 0.001)) (see *figure 3A*).

### AR(1)-model by hospital type

**Figure 4:**
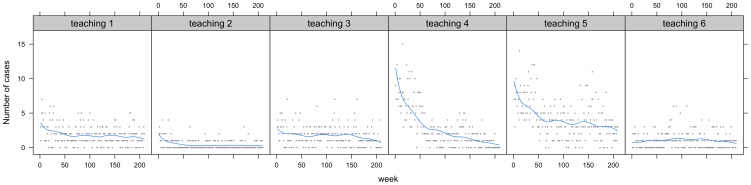
AR(1) model fit teaching hospitals. Grey dots represent the weekly-observed CDI cases within the teaching hospitals from April 2008 to March 2012. X-axis: Week 0 corresponds to the first week of April 2008 and week 209 to the last week of March 2012. Blue line: fit of the AR(1) model with a cubic representation of the rate of change of CDI over time and seasonality.

The negative correlation presented in the AR(1) cubic model after week 20 (*see* *figure 3A*) implied that the model might be over-fitting our data. Also, diagnostic plots suggested deviation from normality in the model's standardized residuals (see *figure S2A*). This can be explained by the large variability in the number of reported cases per hospital, with a much greater number of reports and related rate of change in reports over time from teaching hospitals compared to just a few cases from specialist and some general hospitals. As a consequence, a hospital-specific term in the model representing the change in CDI reports over time might not be suitable for hospitals with only a few cases reported, whereas such specification is required to represent the CDI trend in teaching hospitals. Fitting the model to the more homogeneous group of teaching hospitals only, revealed a stronger, but still relatively low statistically significant correlation between CDI cases and patients present in the hospital one week later (Φ = 0.104 (95% CI = 0.048-0.159) (*see* *figure 2B*), which was captured by the AR(1)-structure (Box-Pierce Q-statistic X^2^  = 23.2, df = 20, p = 0.281) *(see* *figure 3B* *)*. *Figure 4* illustrates the cubic AR(1) model predictions in comparison to the observed teaching hospital data and *figure S2* the model diagnostics.

## Discussion

In this study we explored the significance of symptomatic patient-to-patient CDI transmission in English hospitals as a source of hospital onset CDI. We found a statistical significant signal of dependence between symptomatic CDI patients spending time in hospital close in time, which suggested symptomatic patient-to-patient transmission of CDI was present. Nonetheless, the low magnitude of correlation between weekly cases in the AR(1) model, implies that the role of symptomatic carriers in CDI-acquisition was not as important as previously supposed. The highest number of CDI cases was reported in teaching hospitals, which corresponded to their overall prevalence of hospital-acquired infections (HAI) being among the highest according to the English National Point Prevalence Survey on HAI [8]. This could be attributed to the more vulnerable case-mix of such hospitals, whom might be more prone to acquiring CDI[21]. Taking CDI reports from the teaching hospitals only, the association between symptomatic carriers was somewhat stronger, but still relatively low. Our findings are in line with recent evidence from whole genome sequencing of 1223 isolates from healthcare (among others from two large acute teaching hospitals, one specialist and one general district hospital) and community onset CDI cases in Oxfordshire, England isolated from 2008 to 2011[7]. Less than 20% of the genetically linked CDI positive cases had documented hospital contact with a symptomatic patient[7]. In addition, 45% of the included CDI cases could not be related to any other symptomatic case (community or healthcare setting) as they were too genetically diverse[7]. Even considering the reported low sensitivity of the toxin EIA test[12] used for CDI identification in the referenced study, the diversity argues for alternative sources of many CDI cases.

Improved infection control, with a primary focus on preventing transmission, such as hand hygiene, isolation of symptomatic cases, and environmental cleaning, might result in lower rates of successful transmission between symptomatic cases following contact[22]. In addition, once a patient comes in contact with *C. difficile* or its spores, the development of CDI is dependent on the disruption of the normal gut flora, primarily due to antibiotic use such as broad-spectrum cephalosporins and quinolones[23], [24]. Nonetheless, this would not explain the origin of symptomatic patients lacking a shared spatial-temporal and/or genetic link. *C. difficile* has been recovered from hospital rooms occupied by both symptomatic and asymptomatic carriers[25]–[27] and its spores can persist in the environment for as long as 20 weeks[28]. Therefore, transmission from contaminated hospital surfaces could suggest symptomatic hospital cases are unrelated, whereas actually indirect-cross infection could have occurred. However, a genetic link would still be found if cases had acquired their infection from the same contaminated hospital surface. If no restrictions were applied to the infectious period, incubation period or length of ward contamination, 27% of the sequenced samples in the earlier mentioned study shared both a genetic and an epidemiological hospital contact [7]. Alternatively, asymptomatic carriers could contaminate hospital surfaces with lower intensity than symptomatic carriers, hence cause acquisition at low frequency, which could potentially explain the wide genomic diversity among cases [29].

Importation of symptomatic and asymptomatic carriers from community-settings such as long-term care facilities (LTCF) has also been suggested as a source for hospital-onset CDI[30], [31]. A population-based study conducted in the United States showed that out of a total of 416 identified CDI cases, 41% had onset of symptoms in the community or within 48 hours after admission and no hospitalisation in the 12 weeks prior to onset[32]. We excluded patients with an onset of CDI <48 hours into admission. This is a frequently used, but arbitrary, cut-off to define community-acquired HAI. Hence it is possible that our data included asymptomatic patients who acquired the bacteria elsewhere, and developed symptoms in the hospital >48 hours after admission. Moreover, in addition to onset within 48 hours into admission, no hospitalisation in the past 12 weeks is an often-used additional requirement for community-acquired CDI [11]. As we did not have information on previous hospitalisation, the possibility exists that cases defined as community-acquired in our data, and were therefore excluded, actually were hospital-acquired cases, i.e. patients who acquired *C. difficile* in their previous stay, but started to develop symptoms after discharge and were re-admitted with symptomatic CDI. Furthermore, approximately 20% of cases with a first occurrence of CDI experience recurrence after discontinuation of treatment[33], [34]. Re-admitted CDI carriers, who resolved their symptoms but remained colonised resulting in a recurrent episode once e.g. put on at risk antibiotics, could be partly responsible for the low correlation between symptomatic carriers. However, considering the known chances of relapse, we do not expect these can be primarily responsible for the results of this study.

Finally, and although not our primary focus, we found evidence of seasonal variations in CDI incidence in our selection of English hospitals, with slightly elevated reports of hospital-associated symptomatic CDI in the winter months. Seasonality has been suggested in relation to increased levels of CDI related antibiotics during the winter months in settings outside of England[15]–[17]. Comparison of variability in antibiotic prescribing patterns within English hospitals with fluctuations in hospital reported CDI incidence would be an interesting area of investigation. Nonetheless, the seasonal component in our model only explained a small proportion of the behaviour of the weekly reported CDI (reflected by a moderate decrease of AIC), and we would like to urge for more research on the presence of seasonal patterns of CDI in England.

This study had several limitations. Firstly, we selected weekly intervals for our analysis. Both the incubation time and infectious period of *C. difficile* have not been quantified with certainty. Studies have suggested that person-to-person transmission occurs primarily within a week (ranging from a median of 1, 4 or 8 days after CDI diagnosis)[5], whereas a median incubation time of 2-3 days[12] to 18–33 days has been proposed[5]. Hence, onset of symptoms following symptomatic patient-to-patient transmission might occur after the one-week time interval, which could have affected the strength of correlation between weekly incidences. Secondly, strains may vary in their pathogenicity[35] and transmissibility[6]. The routinely collected surveillance data did not contain ribotype specific information, so we could not establish to what extent our results are strain-specific as well as whether the hospitals in our sample are representative with regards to strain prevalence. Moreover, the AR1 structure was unable to fully explain the correlation between weekly cases close in time using data from all hospital types, whereas it could for the teaching hospitals only. This might be a consequence of the stochastic nature of the few CDI cases reported by the smaller hospitals included in the overall dataset. Alternatively, teaching hospitals might have better environmental cleaning practices in place and/or are more likely to change antibiotic prescribing practices following an outbreak, resulting in more rapid containment. Further research is needed to clarify the observed heterogeneity in reported hospital-acquired infection rates and transmission between teaching and non-teaching hospitals. Finally, alternative causes of dependence of the weekly CDI observations cannot be ruled out, e.g. a Scottish study[36] identified a temporal correlation between antibiotic use and HA-CDI [36]. However, as the results of the referenced study[36] suggest, it is unlikely that antibiotic hospital consumption will fluctuate between weekly time intervals. After investigation of the association between monthly variations in antibiotic use and monthly variations in observed CDI, Vernaz and colleagues (2009) identified that, for almost all of the antibiotics investigated, the association with observed CDI was significant with a lag of several months (among others ciprofloxacin, fluoroquinolones and cefuroxime) [36]. In addition, cases arising from asymptomatic carriers or environmental sources might correlate in space and time as well. However, the level of onward transmission from asymptomatically colonised individuals is highly uncertain, nor has foodborne transmission of *C. difficile* to humans been established with certainty [37], [38]. Given the infectious nature of symptomatic *C. difficile* cases, especially in settings with high antibiotic use, we expect symptomatic patient-to-patient transmission to be the most conservative explanation.

Despite the limited information present in routinely collected hospital infection data, this study has provided further insight in the hospital transmission dynamics of *C. difficile.* Our results indicate that patient-to-patient transmission when only those patients with symptomatic CDI are considered, may account for a small number of transmission events. To improve our understanding of the epidemiology of CDI, the role of other patient groups should be considered, such as those in the community and asymptomatic carriers, as well as the importance of indirect transmission from contaminated surfaces in the hospital environment and the role of antibiotic use. Individual-level patient data, which can inform dynamic transmission models would certainly aid the investigating and quantification of the potential sources of CDI transmission[39]–[41] and will be another area of our further investigations.

## Supporting Information

Figure S1 **Seasonal variations of symptomatic** ***C. difficile*** **infection with onset >48 hours after admission.** Fitted cyclic penalised cubic regression spline (representing seasonal variations) for the cubic AR(1) model fitted to data of all hospitals (A) and data of the teaching hospitals only (B).
(TIF)

Figure S2 **Diagnostic plots cubic AR(1)-models.** A: Residual diagnostics of AR(1)-model fitted to data of all hospital, including a fitted cubic representation of CDI behaviour over time and seasonality. A: Quantile-Quantile (Q-Q) plot, deviation from a straight line denotes deviation from normal distribution, B: residuals plotted against linear predictor; C: frequency distribution of the model residuals; D: data against fitted values. E-H: Residual diagnostics of AR(1)-model fitted to data of teaching hospital only.
(TIF)

Material S1 **Model description and fitting procedure.** Further description of the model used, and model fitting and selection procedure.
(DOCX)

Table S1 **Comparison of model fit for the base-model including alternative representations of the CDI incidence trend.** AIC = Akaike Information criterion, the lower the value, the better the fit. Upper half of table: model fit to data of all hospitals. Lower half: model fit to data from teaching hospitals. Left half of table: model fit assuming a Poisson distribution. Right half: model fit assuming an overdispersed Poisson (quasi-Poisson) distribution.
(DOCX)

## References

[1] Ghantoji SS, Sail K, Lairson DR, DuPont HL, Garey KW (2010) Economic healthcare costs of Clostridium difficile infection: a systematic review. Journal of Hospital Infection 74: 309–318. 20153547 10.1016/j.jhin.2009.10.016

[2] Dubberke ER, Olsen MA (2012) Burden of Clostridium difficile on the Healthcare System. Clinical Infectious Diseases 55: S88–S92 10.1093/cid/cis335 22752870 PMC3388018

[3] Public Health England (former Health Protection Agency) (2012) Mandatory surveillance of Clostridium difficile. PHE website. Available: http://www.hpa.org.uk/web/HPAweb&HPAwebStandard/HPAweb_C/1179746015058. Accessed 2013 Mar 20.

[4] Wilcox MH, Shetty N, Fawley WN, Shemko M, Coen P, et al (2012) Changing Epidemiology of Clostridium difficile Infection Following the Introduction of a National Ribotyping-Based Surveillance Scheme in England. Clinical infectious diseases 55 10.1093/cid/cis614 22784871

[5] Walker a S, Eyre DW, Wyllie DH, Dingle KE, Harding RM, et al (2012) Characterisation of Clostridium difficile Hospital Ward-Based Transmission Using Extensive Epidemiological Data and Molecular Typing. PLoS medicine 9: e1001172 10.1371/journal.pmed.1001172 22346738 PMC3274560

[6] Didelot X, Eyre D, Cule M, Ip C, Ansari A, et al (2012) Microevolutionary analysis of Clostridium difficile genomes to investigate transmission. Genome Biology 13: R118 10.1186/gb-2012-13-12-r118 23259504 PMC4056369

[7] Eyre DW, Cule ML, Wilson DJ, Griffiths D, Vaughan A, et al (2013) Diverse Sources of C. difficile Infection Identified on Whole-Genome Sequencing. New England Journal of Medicine 369: 1195–1205 10.1056/NEJMoa1216064 24066741 PMC3868928

[8] Public Health England (former Health Protection Agency) (2012) English National Point Prevalence Survey on Healthcare-associated Infections and Antimicrobial Use, 2011 - preliminary data. London.

[9] Goldenberg SD, French GL (2011) Diagnostic testing for Clostridium difficile: a comprehensive survey of laboratories in England. The Journal of hospital infection 79: 4–7 10.1016/j.jhin.2011.03.030 21724296

[10] Lewis S, Heaton K (1997) Stools form scale as a usefule guide to intestinal transit time. Scandinavian Journal of Gastroenterology 32: 920–924. 9299672 10.3109/00365529709011203

[11] Department of Health (2012) Updated guidance on the diagnosis and reporting of Clostridium difficile.

[12] Cohen SH, Gerding DN, Johnson S, Kelly CP, Loo VG, et al (2010) Clinical practice guidelines for Clostridium difficile infection in adults: 2010 update by the society for healthcare epidemiology of America (SHEA) and the infectious diseases society of America (IDSA). Infection control and hospital epidemiology 31: 431–455 10.1086/651706 20307191

[13] Goldstein H (2010) Multilevel Statistical Models. 4th ed. Chichester: Wiley-Blackwell.

[14] Snijder TAB, Bosker RJ (2012) Multilevel analysis: An introduction to basic and advanced multilevel modeling. 2nd ed. London, England: SAGE publications.

[15] Archibald LK, Banerjee SN, Jarvis WR (2004) Secular trends in hospital-acquired Clostridium difficile disease in the United States, 1987–2001. The Journal of infectious diseases 189: 1585–1589. 15116293 10.1086/383045

[16] Polgreen P, Yang M, Bohnett L, Cavanaugh J (2010) A Time-Series Analysis of Clostridium difficile and Its Seasonal Association with Influenza. Infection Control Hosp Epidemiol 31: 382–387 10.1086/651095.A PMC3024857 20175682

[17] Gilca R, Fortin E, Frenette C, Longtin Y, Gourdeau M (2012) Seasonal variations in Clostridium difficile infections are associated with influenza and respiratory syncytial virus activity independently of antibiotic prescriptions: a time series analysis in Quebec, Canada. Antimicrobial agents and chemotherapy 56: 639–646 10.1128/AAC.05411-11 22106208 PMC3264229

[18] Wood SN (2006) Generalized Additive Models: an introduction with R. 1st ed. London: Chapman and Hall/CRC.

[19] Pinheiro JC, Bates DM (2000) Mixed-Effects Models in S and S-PLUS. New York: Springer.

[20] Intelligence dr F (2013) Hospital guide. Available: http://www.drfosterhealth.co.uk/hospital-guide/.

[21] Söderlund N, Milne R, Gray A, Raftery J (1995) Differences in hospital casemix, and the relationship between casemix and hospital costs. Journal of public health medicine 17: 25–32. 7786563

[22] Hsu J, Abad C, Dinh M, Safdar N (2010) Prevention of endemic healthcare-associated Clostridium difficile infection: reviewing the evidence. American Journal of Gastroenterology 105: 2327–39 quiz 2340. 20606676 10.1038/ajg.2010.254

[23] Starr JM, Martin H, McCoubrey J, Gibson G, Poxton IR (2003) Risk factors for Clostridium difficile colonisation and toxin production. Age and ageing 32: 657–660. 14600008 10.1093/ageing/afg112

[24] Thomas C, Stevenson M, Riley TV (2003) Antibiotics and hospital-acquired Clostridium difficile-associated diarrhoea: a systematic review. Journal of Antimicrobial Chemotherapy 51: 1339–1350. 12746372 10.1093/jac/dkg254

[25] McFarland LV, Mulligan ME, Kwok RYY, Stam WE (1989) Nosocomial acquisition of Clostridium difficile infection. New England Journal of Medicine 321: 190. 10.1056/NEJM198901263200402 2911306

[26] Samore MH, Venkataraman L, Degirolami PC, Arbeit RD, Karchmer AW (1996) Clinical and Molecular Epidemiology of Sporadic and Clustered Cases of Nosocomial Clostridium difficile Diarrhea. the American Journal of Medicine 100: 32–40. 8579084 10.1016/s0002-9343(96)90008-x

[27] Kaatz G, Gitlin S, Schaberg D, Wilson KH, Kauffman CA, et al (1988) Acquisition of clostridium difficile from the hospital environment. American Journal of Epidemiology 127: 1289–1294. 2835900 10.1093/oxfordjournals.aje.a114921

[28] Kim K, Fekety R, Batts DH, Brown D (1981) Isolation of Clostridium difficile from the Environment and Contacts of Patients with Antibiotic-Associated Colitis. The Journal of infectious diseases 143: 42–50. 7217711 10.1093/infdis/143.1.42

[29] Guerrero DM, Becker JC, Eckstein EC, Kundrapu S, Deshpande A, et al (2013) Asymptomatic carriage of toxigenic Clostridium difficile by hospitalized patients. The Journal of hospital infection: 2–5. 10.1016/j.jhin.2013.07.002 23954113

[30] Ricciardi R, Nelson J, Griffith JL, Concannon TW (2012) Do admissions and discharges to long-term care facilities influence hospital burden of Clostridium difficile infection? The Journal of hospital infection 80: 156–161 10.1016/j.jhin.2011.11.002 22137065 PMC3262915

[31] Riggs MM, Sethi AK, Zabarsky TF, Eckstein EC, Jump RLP, et al (2007) Asymptomatic Carriers Are a Potential Source for Transmission of Epidemic and Nonepidemic Clostridium difficile Strains among Long-Term Care Facility Residents. Clinical Infectious Diseases 45: 992–998 10.1086/521854 17879913

[32] Khanna S, Pardi DS, Aronson SL, Kammer PP, Orenstein R, et al (2011) The Epidemiology of Community-Acquired Clostridium diffi cile Infection: A Population-Based Study. The American Journal of Gastroenterology 107: 89–95 10.1038/ajg.2011.398 22108454 PMC3273904

[33] Kamboj M, Khosa P, Kaltsas A, Babady NE, Son C, et al (2011) Relapse versus reinfection: surveillance of Clostridium difficile infection. Clinical infectious diseases 53: 1003–1006 10.1093/cid/cir643 21976462 PMC3246877

[34] Fekety R, McFarland L V, Surawicz CM, Greenberg RN, Elmer GW, et al (1997) Recurrent Clostridium difficile diarrhea: characteristics of and risk factors for patients enrolled in a prospective, randomized, double-blinded trial. Clinical infectious diseases: an official publication of the Infectious Diseases Society of America 24: 324–333. 9114180 10.1093/clinids/24.3.324

[35] Walker AS, Eyre DW, Wyllie DH, Dingle KE, Griffiths D, et al (2013) Relationship Between Bacterial Strain Type, Host Biomarkers, and Mortality in Clostridium difficile Infection. Clinical infectious diseases 56: 1589–1600 10.1093/cid/cit127 23463640 PMC3641870

[36] Vernaz N, Hill K, Leggeat S, Nathwani D, Philips G, et al (2009) Temporal effects of antibiotic use and Clostridium difficile infections. The Journal of antimicrobial chemotherapy 63: 1272–1275 10.1093/jac/dkp128 19372170

[37] Eyre DW, Griffiths D, Vaughan A, Golubchik T, Acharya M, et al (2013) Asymptomatic Clostridium difficile colonisation and onward transmission. PloS one 8: e78445 10.1371/journal.pone.0078445 24265690 PMC3827041

[38] Gould LH, Limbago B (2010) Clostridium difficile in food and domestic animals: a new foodborne pathogen? Clinical infectious diseases: an official publication of the Infectious Diseases Society of America 51: 577–582 10.1086/655692 20642351

[39] Bootsma MCJ, Bonten MJM, Nijssen S, Fluit a C, Diekmann O (2007) An algorithm to estimate the importance of bacterial acquisition routes in hospital settings. American journal of epidemiology 166: 841–851 10.1093/aje/kwm149 17644823

[40] McBryde ES, Pettitt AN, Cooper BS, McElwain DLS (2007) Characterizing an outbreak of vancomycin-resistant enterococci using hidden Markov models. Journal of the Royal Society Interface 4: 745–754. 10.1098/rsif.2007.0224 PMC2373397 17360254

[41] Forrester ML, Pettitt AN, Gibson GJ (2007) Bayesian inference of hospital-acquired infectious diseases and control measures given imperfect surveillance data. Biostatistics 8: 383–401. 16926230 10.1093/biostatistics/kxl017

