# Supplementary figures and images for: Nosocomial Transmission of C. difficile in English Hospitals from Patients with Symptomatic Infection
Source: PLoS One. 2014 Jun 16;9(6):e99860. doi: 10.1371/journal.pone.0099860 (PMC4059673; doi:10.1371/journal.pone.0099860)

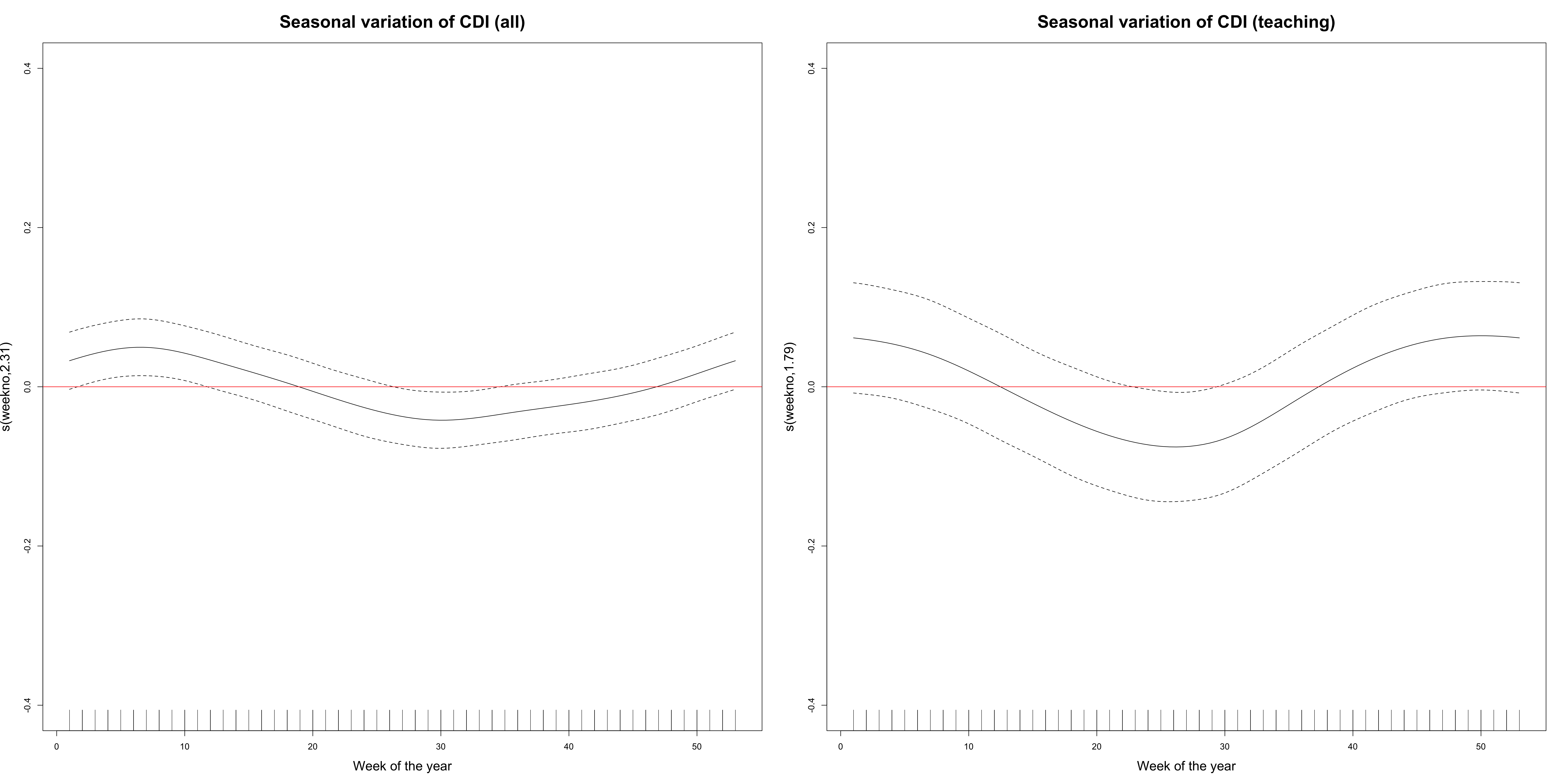

Supplement: Figure S1 — Seasonal variations of symptomatic C. difficile infection with onset >48 hours after admission. Fitted cyclic penalised cubic regression spline (representing seasonal variations) for the cubic AR(1) model fitted to data of all hospitals (A) and data of the teaching hospitals only (B). (TIF) [file pone.0099860.s001.tif]

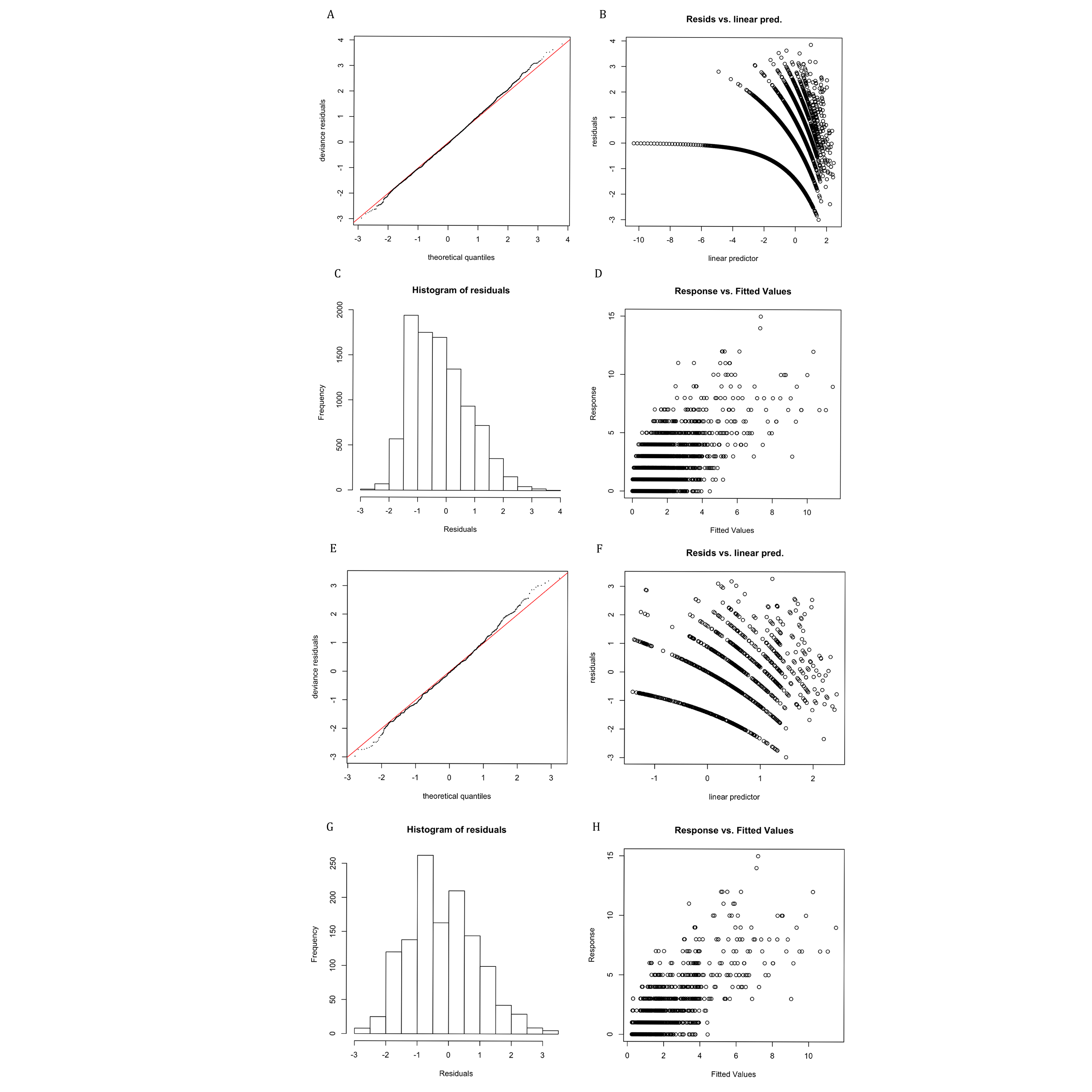

Supplement: Figure S2 — Diagnostic plots cubic AR(1)-models. A: Residual diagnostics of AR(1)-model fitted to data of all hospital, including a fitted cubic representation of CDI behaviour over time and seasonality. A: Quantile-Quantile (Q-Q) plot, deviation from a straight line denotes deviation from normal distribution, B: residuals plotted against linear predictor; C: frequency distribution of the model residuals; D: data against fitted values. E-H: Residual diagnostics of AR(1)-model fitted to data of teaching hospital only. (TIF) [file pone.0099860.s002.tif]
