# Supplementary material for: Nosocomial Transmission of C. difficile in English Hospitals from Patients with Symptomatic Infection
Source: PLoS One. 2014 Jun 16;9(6):e99860. doi: 10.1371/journal.pone.0099860 (PMC4059673; doi:10.1371/journal.pone.0099860)
Supplement: Material S1 — Model description and fitting procedure. Further description of the model used, and model fitting and selection procedure. (DOCX) [file pone.0099860.s003.docx]

Materials S1

**A1. Model description**

The following generalized additive mixed effects Poisson model allowing for overdispersion, with a log link[1,2] was used for fitting to the data of all hospitals as well as of the teaching hospitals alone:

log($\lambda_{jt})=\alpha_{j}+f_{1}(t)+f_{2}(t_{w.o.y.})+R_{jt}$

Where, $\lambda_{jt}$ indicates the observed number of CDI cases $\lambda$ at week *t* in individual hospital *j,* given the random error term $R_{jt}$ (see later), $\alpha_{j}$and $f_{1}(t)$ are the individual hospital intercept and representation of the cross-sectional trend respectively, and $f_{2}\left( t_{w.o.y.} \right)$ the seasonal variation in CDI incidence. The alternative representations of the longitudinal CDI trend were specified as a linear, quadratic and cubic polynomial respectively. We added a cyclic (periodic) penalised cubic regression spline over the variable week of the year, i.e. $f_{2}\left( t_{w.o.y.} \right)= \sum_{i=1}^{k-1} \tilde{b_{i}}\left( t_{w.o.y.} \right)S_{i}$; we provided the end points of the smoother (i.e. the first and last week of the year, week 1 and week 53) and allowed automatic generation of the remainder knots (knots specify adjacent intervals, where each interval represents an individual polynomial)[3]. Finally, $R_{jt}$ denotes the residual term in hospital *j* at week *t*, which were of primary interest. We assumed a temporal proximity pattern, i.e. incidences of weeks close in time are strongly correlated, and that this correlation faded rapidly over time according to $R_{jt}={\phi^{k}R}_{j (t-k)}, k=1, 2, \ldots$.[4], *k* represents the weekly distance between the incidence observations and $\phi$ corresponds to the strength of the correlation, i.e. the correlation coefficient. If $\phi^{k}$= 0, this corresponds to no (auto)correlation, and thus no weekly CDI dependence, indicating no symptomatic transmission.

**A2. Model Fitting**

The base-model (assuming no transmission, i.e. excluding AR(1)) was fitted by a penalised likelihood using the command gam() (library mgcv). The different polynomials, denoting alternative representations of the CDI incidence trend, were fitted by generating a B-spline basis matrix without interior knots using the command bs()(library splines) and seasonality was represented by a cyclic cubic smooth function using the command s(.., bs = “cc”) (library mgcv)[3]. The models were fitted both with a Poisson and quasi-Poisson distribution, the latter to allow for overdispersion (represented by a scale parameter >1 (*see table S1*), which is common in hospital count data. For all three representations of the base-model, a model including seasonal patterns provided a moderately better fit (with a reduction in AIC of 15.3, 14.3, 16.3, for the linear, quadratic and cubic null-models including seasonality, respectively). For the seasonal trend, the distributional assumption under the null-hypothesis (chi-square) does not have a firm theoretical basis and is conditional on the smoothing parameter (i.e. the degrees of freedom estimated for the smooth term). Hence, the p-value provided by the gam model for the seasonal trend is an approximation and should be considered with care[3]. However, considering the reduction in AIC, seasonality was kept in the model. *Table S1* summarizes model fit of the different representations of the declining incidence trend over time and adjusting for seasonality. A linear representation provided the worst model fit. Both the quadratic and the cubic time trend showed a considerable improvement in model fit in comparison to the linear trend, while the cubic time trend showed a moderately better fit in comparison to the quadratic time trend. As a next step, we added an AR(1) temporal covariance structure using the Penalised Quasi-likelihood based (PQL) based command gamm()(library mgcv). In contrast to gam(), this command allows the addition of patterned covariance structures. This method can work poorly (i.e. underestimate the standard error of the fitted parameters) for Poisson data with a mean number of counts of less than five[1,5]. Comparison of model fit by a penalised likelihood and PQL resulted in similar model estimates for our base-model, which provided confidence in the PQL method used for our data. Another caveat of the PQL method is the lack of a real likelihood. Hence, it was not possible to formally test whether the inclusion of the AR(1) residual correlation structure indeed did improve model fit. For this reason, we evaluated the estimated value of $\phi^{k}$ (i.e. whether departure from 0), and the autocorrelation function (ACF) coefficients of the best fitting null-model and AR1 model normalized residuals to test departure from independence (p <0.05).

**A4. References**

1. Snijder TAB, Bosker RJ (2012) Multilevel analysis: An introduction to basic and advanced multilevel modeling. 2nd ed. London, England: SAGE publications.

2. Goldstein H (2010) Multilevel Statistical Models. 4th ed. Chichester: Wiley-Blackwell.

3. Wood SN (2006) Generalized Additive Models: an introduction with R. 1st ed. London: Chapman and Hall/CRC.

4. Pinheiro JC, Bates DM (2000) Mixed-Effects Models in S and S-PLUS. New York: Springer.

5. Bolker BM, Brooks ME, Clark CJ, Geange SW, Poulsen JR, et al. (2009) Generalized linear mixed models: a practical guide for ecology and evolution. Trends Ecol Evol 24: 127–135. doi:10.1016/j.tree.2008.10.008.
