# Supplementary material for: Nosocomial Transmission of C. difficile in English Hospitals from Patients with Symptomatic Infection
Source: PLoS One. 2014 Jun 16;9(6):e99860. doi: 10.1371/journal.pone.0099860 (PMC4059673; doi:10.1371/journal.pone.0099860)
Supplement: Table S1 — Comparison of model fit for the base-model including alternative representations of the CDI incidence trend. AIC = Akaike Information criterion, the lower the value, the better the fit. Upper half of table: model fit to data of all hospitals. Lower half: model fit to data from teaching hospitals. Left half of table: model fit assuming a Poisson distribution. Right half: model fit assuming an overdispersed Poisson (quasi-Poisson) distribution. (DOCX) [file pone.0099860.s004.docx]

**Table SI: Comparison of model fit for the base-model including alternative representations of the CDI incidence trend**

| All hospitals |  | Poisson |  | |  | | |  | |  |  | Quasi-Poisson | | |  | |  | | | |  | | |  | | |  | |  | | |
| --- | --- | --- | --- | --- | --- | --- | --- | --- | --- | --- | --- | --- | --- | --- | --- | --- | --- | --- | --- | --- | --- | --- | --- | --- | --- | --- | --- | --- | --- | --- | --- |
| Model |  | **AIC** |  | | **X^2^** | | |  | | **p-value seasonality** |  | **AIC** |  | | | **F** | | | |  | | | **p-value seasonality** | | |  | | **Scale parameter** | | |  |
| *Linear + seasonality* |  | 22544 |  | | 25.91 | | |  | | .0002 |  | NA |  | | | 25.91 | | | |  | | | .0006 | | |  | | 1.095 | | |  |
|  |  |  |  | |  | | |  | |  |  |  |  | | |  | | | |  | | |  | | |  | |  | | |  |
| *Quadratic + seasonality* |  | 22454 |  | | 24.99 | | |  | | .0003 |  | NA |  | | | 2.837 | | | |  | | | .0008 | | |  | | 1.081 | | |  |
|  |  |  |  | |  | | |  | |  |  |  |  | | |  | | | |  | | |  | | |  | |  | | |  |
| *Cubic + seasonality* |  | 22426 |  | | 27.31 | | |  | | .0001 |  | NA |  | | | 3.131 | | | |  | | | .0003 | | |  | | 1.074 | | |  |
|  |  |  |  | |  | | |  | |  |  |  |  | | |  | | | |  | | |  | | |  | |  | | |  |
| *Cubic no seasonality* |  | 22442 |  | | - | | |  | | - |  | NA |  | | | - | | | |  | | | - | | |  | | 1.076 | | |  |
|  |  |  |  | |  | | |  | |  |  |  |  | | |  | | | |  | | |  | | |  | |  | | |  |
| Teaching hospitals |  | **Poisson** | |  | |  |  | |  | |  | **Quasi-Poisson** | |  | | | |  |  | | |  | | |  | | |  | |  |  |
| Model |  | **AIC** | |  | | **X^2^** |  | | **p-value seasonality** | |  | **AIC** | |  | | | | **F** |  | | | **p-value seasonality** | | |  | | | **Scale parameter** | | |  |
| *Linear + seasonality* |  | 4054 |  | | 6.54 | | |  | | .142 |  | NA |  | | | .003 | | | |  | | | .374 | | |  | | 1.304 | | |  |
|  |  |  |  | |  | | |  | |  |  |  |  | | |  | | | |  | | |  | | |  | |  | | |  |
| *Quadratic + seasonality* |  | 4032 |  | | 6.33 | | |  | | .108 |  | NA |  | | | .156 | | | |  | | | .222 | | |  | | 1.282 | | |  |
|  |  |  |  | |  | | |  | |  |  |  |  | | |  | | | |  | | |  | | |  | |  | | |  |
| *Cubic + seasonality* |  | 4018 |  | | 18.18 | | |  | | .005 |  | NA |  | | | .854 | | | |  | | | .016 | | |  | | 1.266 | | |  |
|  |  |  |  | |  | | |  | |  |  |  |  | | |  | | | |  | | |  | | |  | |  | | |  |
| *Cubic no seasonality* |  | 4025 |  | | - | | |  | | - |  | NA |  | | | - | | | |  | | | - | | |  | | 1.273 | | |  |

AIC = Akaike Information criterion, the lower the value, the better the fit. Upper half of table: model fit to data of all hospitals. Lower half: model fit to data from

teaching hospitals. Left half of table: model fit assuming a Poisson distribution. Right half: model fit assuming an overdispersed Poisson (quasi-Poisson) distribution.
